# Migration and Proliferation Effects of Thymoquinone-Loaded Nanostructured Lipid Carrier (TQ-NLC) and Thymoquinone (TQ) on *In Vitro* Wound Healing Models

**DOI:** 10.1155/2019/9725738

**Published:** 2019-11-29

**Authors:** Henna Roshini Alexander, Sharifah Sakinah Syed Alwi, Latifah Saiful Yazan, Fatin Hanani Zakarial Ansar, Yong Sze Ong

**Affiliations:** Department of Biomedical Science, Faculty of Medicine and Health Sciences, Universiti Putra Malaysia (UPM), 43400 Serdang, Selangor, Malaysia

## Abstract

Wound healing is a regulated biological event that involves several processes including infiltrating leukocyte subtypes and resident cells. Impaired wound healing is one of the major problems in diabetic patients due to the abnormal physiological changes of tissues and cells in major processes. Thymoquinone, a bioactive compound found in *Nigella sativa* has been demonstrated to possess antidiabetic, anti-inflammatory, and antioxidant effects. Today, the rapidly progressing nanotechnology sets a new alternative carrier to enhance and favour the speed of healing process. In order to overcome its low bioavailability, TQ is loaded into a colloidal drug carrier known as a nanostructured lipid carrier (NLC). This study aimed to determine the effect of TQ-NLC and TQ on cell proliferation and migration, mode of cell death, and the antioxidant levels in normal and diabetic cell models, 3T3 and 3T3-L1. Cytotoxicity of TQ-NLC and TQ was determined by MTT assay. The IC_10_ values obtained for 3T3-L1 treated with TQ-NLC and TQ for 24 hours were 4.7 ± 3.3 and 5.3 ± 0.6 *μ*M, respectively. As for 3T3, the IC_10_ values obtained for TQ-NLC and TQ at 24 hours were 4.3 ± 0.17 and 3.9 ± 2.05 *μ*M, respectively. TQ-NLC was observed to increase the number of 3T3 and 3T3-L1 healthy cells (87–95%) and gradually decrease early apoptotic cells in time- and dose-dependant manner compared with TQ. In the proliferation and migration assay, 3T3-L1 treated with TQ-NLC showed higher proliferation and migration rate (*p* < 0.05) compared with TQ. TQ-NLC also acted as an antioxidant by reducing the ROS levels in both cells after injury at concentration as low as 3 *μ*M. Thus, this study demonstrated that TQ-NLC has better proliferation and migration as well as antioxidant effect compared with TQ especially on 3T3-L1 which confirms its ability as a good antidiabetic and antioxidant agent.

## 1. Introduction

Wounds are physical insults that result in a break or opening of the skin [1]. They can be produced by physical, chemical, or microbial insult to the tissue [2] and are a major cause of physical disability [3]. Wounds are generally classified into two types: acute and chronic wound. Acute wounds usually repair themselves in an orderly manner which causes both functional and anatomical restoration [4]. Any alterations that interrupt the timely controlled healing processes would prolong tissue damage and the repair process, consequently contributing to chronic wound [5], and complications usually entail. Chronic wounds are wounds that display impaired healing. They usually have failed to progress through the normal stages of healing [6] and get trapped in a permanent inflammatory stage due to an imperfect or uncoordinated healing process [7–9]. Wound healing is the body's natural reaction that leads to repair of the injured tissue [10] and is not seen as a problem in normal healthy individuals [11]. Many of the repair processes are common to all tissues and take place starting at the cellular stage. Proper levels of cytokines and growth factors are needed for proper healing of the wound [12].

The process of wound healing comprises four highly integrated and overlapping phases (haemostasis, inflammation, proliferation, and remodelling of tissue) [13, 14]. It includes the coordinated effort of several cell types such as fibroblasts, platelets, macrophages, keratinocytes, and endothelial cells. This complex process is carried out by a signalling network comprising various cytokines, growth factors, and chemokines [15]. Each phase's length may vary depending on the physiological and exogenous factors affecting the wound. Proliferation phase is the most important repair process in wound healing [16]. The chief cells of this phase are the epithelial cells. Epithelial cell migration is a complex but dynamic process of wound healing that plays a critical role in health and disease, including embryogenesis, immune response, and tissue development. It requires the coordination of numerous cellular processes [17]. The reepithelialization phase is where cell migration mainly takes place, and it plays an important role in angiogenesis, which provides oxygen and nutrients to the repairing tissue. When the cells migrate, they help restore the functional integrity of the epithelial barriers breached by insult. Consequently, inefficient cell migration will result in impaired wound healing [18]. When skin is wounded, blood clot immediately forms within seconds, and immune cells infiltrate the wound site. In order for wound healing to take place, the fibrin clot formed must be destroyed to facilitate cell migration to the wound site.

During the proliferative stage, the cells in the epidermis and dermis start proliferating and migrating into the wound bed. Then, dermal cells are deposited and continue restructuring the ECM in the wound bed until the wound is closed [19]. Fibroblasts are a heterogeneous cell population responsible for matrix production and remodelling and are required to migrate and proliferate at the wound site for granulation tissue formation as they help in the development of the extracellular matrix [20]. *Nigella sativa* Linn., (*N*. *sativa*) is an annual flowering plant belonging to dicotyledon of the Ranunculaceae family [21]. It is an amazing herb with a rich historical background [22]. It is native to North Africa, Southern Europe, and Southwest Asia. It is vastly cultivated in many countries such as the Middle Eastern Mediterranean region, Syria, India, Turkey, Pakistan, southern Europe, and Saudi Arabia [23]. The Arabic name of *N*. *sativa* is Habbatus Sauda. It is also known by other names such as black caraway, roman coriander, kalonji, and kezah [24]. The seed of *N*. *sativa*, known as black seed, has been used for centuries for culinary [25] and medicinal purposes [26, 27]. It is used as a spice and condiment [28, 29] and was one of the earliest cultivated plants in the human history. It also has been utilized as herbal medicine by various cultures to treat various ailments [30, 31] including high blood pressure, headache, fever, dizziness, and influenza [32, 33]. The principle active ingredient isolated from the essential oil of *N*. *sativa* is thymoquinone (TQ) [34, 35]. TQ is responsible for the therapeutic properties of this plant [23].

The pharmacological investigation of the seed extracts reveals a variety of activities including antihypertensive [36], anti-inflammatory [28, 37], antihistaminic [38], antimicrobial [39, 40], antioxidant [41], and antidiabetic [42, 43]. Many studies on *N*. *sativa* and TQ have been recorded before to find a solution for diabetes mellitus, yet none have been carried out to observe the effect of TQ on wounds in diabetic patients. Studies on *N*. *sativa* effects on healing burn wounds have been conducted by Yaman and Balikci [44] and Sarkhail et al. [45] before. Although TQ is known for its varied functions, because its bioavailability is poor, its clinical use is limited [46]. Therefore, to overcome this problem, together with the rapidly progressing nanotechnology today, it is indeed important to look at a new alternative that has been encapsulated with a carrier that may enhance and reach beneath the skin to effectively speed the healing process.

Nanostructured lipid carrier (NLC), a colloidal drug carrier, is capable of increasing the bioavailability of poorly soluble drugs, facilitate controlled release of drugs, and provide protection for sensitive active compounds or drugs [47]. As the first generation nanoparticles came with many drawbacks, NLC, a second generation nanoparticle was developed with improved features, such as small partice size and a better drug loading capacity. It is produced by combining the solid and liquid lipid together as this increases the loading capacity of any drug of interest [48]. As Ng et al. [49] previously described, thymoquinone-loaded nanostructured lipid carrier (TQ-NLC) (patent no: PI201200181) was produced through a hot high-pressure homogenization method and yielded remarkable physicochemical properties. With high encapsulation efficiency and a particle size of less than 50 nm in diameter, TQNLC has a stability of up to two years [49]. Due to its small particle size, it presents a larger surface area for reaction with its target component and also minimizes the probability of it being phagocytosed by macrophages [50, 51]. TQ-NLC has a huge potential to be used for wounds of diabetic patients. With all the attributes of NLC and the effectiveness of TQ as an antioxidant, anti-inflammation, and antidiabetic agent, TQ-NLC may be an effective agent for diabetic wound healing.

## 2. Materials and Methods

### 2.1. Chemicals and Reagents

Dulbecco's Modifed Eagle's Medium (DMEM), antibiotics (penicillin and streptomycin), trypan blue dye solution, and trypsin-EDTA were purchased from Nacalai Tesque (Kyoto, Japan). Phosphate buffer saline (PBS) tablets and 3-(4,5-dimethylthiazol-2-yl)-2,5 diphenyltetrazolium bromide (MTT) powder were purchased from Merck (Darmstadt, Germany), and dimethyl sulfoxide (DMSO) was purchased from Fisher Sc. (UK). Fetal bovine serum (FBS) was purchased from JR Scientific Inc. (California, USA). Annexin V/FITC kit was purchased from BD Bioscience (USA). Assoc. Prof. Dr. Latifah Saiful Yazan from the Laboratory of Molecular Biomedicine, Institute of Bioscience (IBS), Universiti Putra Malaysia provided TQ and TQ-NLC.

### 2.2. Cell Lines

Mouse embryonic fibroblast (NIH/3T3) and mouse fibroblast diabetic cell model and 3T3-L1 cell lines were obtained from the American Type Culture Collection (Rockville, USA).

### 2.3. Cell Viability Assay

Cytotoxicity of TQ-NLC and TQ was determined using MTT 3-(4,5 dimethylthiazol2-yl)-2,5-diphenyltetrazolium bromide (Merck, Darmstadt, Germany) assay. Briefly, 4.0 × 10^4^ cells in media with 10% serum were seeded and grown in a 96-well microtiter plate (100 *μ*L culture medium per well) and incubated for 24 hours at 37°C in a 5% carbon dioxide incubator to allow the cells to adhere to the plastic substratum. The cells were then treated with TQ-NLC and TQ at various concentrations. Untreated cells were used as control. The cells were incubated in a 5% carbon dioxide incubator at 37°C for 24, 48, and 72 hours followed with addition of 5 mg/ml of MTT solution into each well. Absorbance at 570 nm and the reference wavelength of 630 nm were measured using a microplate reader (Opsys MR, USA).

### 2.4. Annexin V Staining

Analysis of mode of cell death was performed by using the Human Annexin V-FITC Apoptosis Detection kit (BD Bioscience, USA). Briefly, 3 × 10^5^ cells in media with 10% serum of 3T3 or 3T3-L1 were seeded into each well of a 6-well plate. After incubation for 24 hours, the cells were treated with TQ-NLC and TQ (3, 6, and 12 *μ*M). The cells were then incubated for 24, 48, and 72 hours. Untreated cells were also included as control. The harvested cells were washed twice with ice-cold PBS and resuspended in 185 *μ*L of 1X binding buffer, 5 *μ*L of annexin V, and 10 *μ*L of propidium iodide (PI) for 10 minutes at room temperature (25°C) in the dark. The number of healthy cells, early apoptotic, late apoptotic, and necrotic cells was quantified by a flow cytometer (Becton Dickinson FACSCalibur, San Jose, CA) [52].

### 2.5. Scratch Wound Healing Assay

Scratch assay was used to evaluate the ability of TQ-NLC and TQ to induce cell migration into the wounded area [53]. Both 3T3 and 3T3-L1 were seeded in a density of 1.0 × 10^5^ cells/well in 6-well plates and allowed to grow to a confluent monolayer. A sterile 200 *μ*l pipette tip was then used to generate scratches of approximately similar sizes in the cell monolayer. Loosened cellular debris was rinsed using phosphate buffer saline (PBS). The scratch test assay was performed in triplicate and was repeated at least three times for each group. The cells were then treated with TQ-NLC and TQ (3, 6, and 12 *μ*M) in serum-free media and compared it with the control group. Images of the cells were taken at a time interval of 0, 12, 24, and 48 hours following treatment with TQ-NLC or TQ. Data were inspected using a phase contrast microscope, and images were captured using a digital camera attached to the microscope and computer system. Data were further analysed using ImageJ imaging software to quantitatively measure the distance travelled by the cells into the wound and the percentage of wound closure at each time point. The percentage of wound closure was calculated as follows:

(1) $$\begin{array}{c} \%\text{ wound closure}=\frac{\left(D_{0}-D_{n}\right)}{D_{0}}\times 100\%, \end{array}$$

where *D*_0_ is the initial distance between both sides of the scratch and *D*_*n*_ is the distance between both sides of the scratch at the measured time.

### 2.6. ROS Assay

Wound healing process can be aided by antioxidants. 3T3 and 3T3-L1 were seeded in a 96-well black plate at a density of 2.5 × 10^4^ cells/well in the complete growth medium. After overnight incubation to allow cell attachment, TQ-NLC and TQ (3, 6, and 12 *μ*M) were added and incubated for 24 hours. To evaluate the oxidative status, cells were then incubated for 30 minutes with 25 *μ*M DCFH-DA (to detect peroxyl radical and hydrogen peroxide) diluted in DMSO at 20 mM. 100 *μ*M H_2_O_2_ was used as a positive control. Principally, the nonpolar and nonionic DCFHDA that crosses the cell membranes will be hydrolyzed to nonfluorescent 2′,7′-dichlorofluorescein (DCFH) by intracellular esterases. The experiment was performed in triplicate.

### 2.7. Statistical Analysis

All data were analysed with two-way analysis of variance (ANOVA) and Dunnett's post hoc test using Graph-Pad Prism 5 (Graph-Pad, La Jolla, CA) software. All data were expressed as mean ± standard error of mean (SEM). Probability of *p* < 0.05 was considered significant.

## 3. Results

### 3.1. Viability Analysis on 3T3 and 3T3-L1

Data observed demonstrated the effect of TQ-NLC or TQ on the viability of 3T3 and 3T3-L1 in dose- and time-dependent manner. The IC_10_ value was selected from the dose-response curve to evaluate the concentrations that can kill 10% or lesser cell population (supplementary materials: Figures [Supplementary-material supplementary-material-1] and [Supplementary-material supplementary-material-1]).  Table 1 shows the IC_10_ values for 3T3 and 3T3-L1 treated with TQ-NLC or TQ. The IC_10_ values obtained for 3T3-treated TQ-NLC for 24, 48, and 72 hours were 4.3 ± 0.17, 3.2 ± 0.54, and 2.5 ± 0.57 *μ*M, respectively, while in 3T3-treated TQ at similar time points, the IC_10_ values were 3.9 ± 2.05, 5.9 ± 1.88, and 4.5 ± 1.44 *μ*M, respectively.

**Figure 1:**
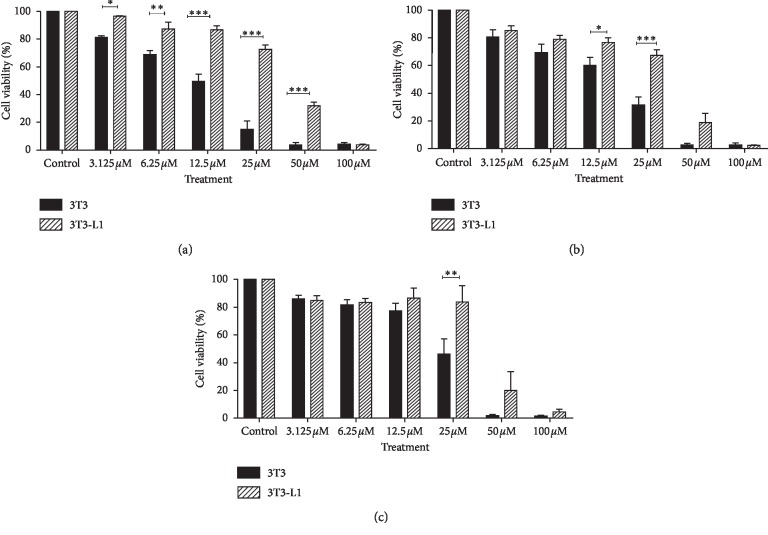
Effect of TQ-NLC on the viability of 3T3 and 3T3-L1. Comparison of the cell viability between 3T3 and 3T3-L1 cells when treated with TQ-NLC at (a) 24 h, (b) 48 h, and (c) 72 h. The data are presented as mean ± SEM. Statistically significant differences are indicated as ^*∗*^*p* < 0.05, ^*∗∗*^*p* < 0.01, and ^*∗∗∗*^*p* < 0.001. Statistically significant difference between 3T3 and 3T3-L1 is also indicated in the graph. Data shown are the average of three independent experiments.

**Figure 2:**
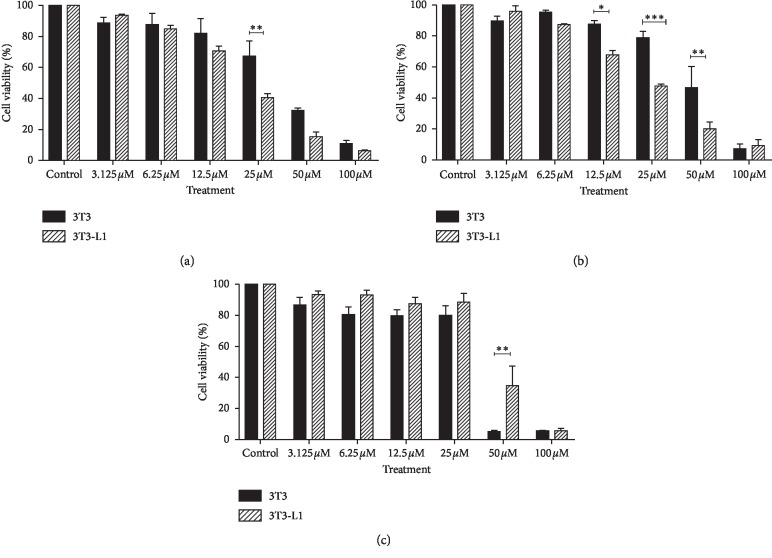
Effect of TQ on the viability of 3T3 and 3T3-L1. Comparison of the cell viability between 3T3 and 3T3-L1 cells when treated with TQ at (a) 24 h, (b) 48 h, and (c) 72 h. The data are presented as mean ± SEM. Statistically significant differences are indicated as ^*∗*^*p* < 0.05, ^*∗∗*^*p* < 0.01, and ^*∗∗∗*^*p* < 0.001. Statistically significant difference between 3T3 and 3T3-L1 is also indicated in the graph. Data shown are the average of three independent experiments.

In 3T3-L1-treated TQ-NLC, the IC_10_ values were 4.7 ± 3.3, 3.5 ± 0.76, and 3.3 ± 0.5 *μ*M, respectively, after 24, 48, and 72 hours of treatment. At lower dosage, TQ-NLC or TQ exhibited similar trend where an increase in cells proliferation was observed. Although both compounds enhanced the proliferation of both normal and diabetic cell models, TQ-NLC induced significant cell proliferation in 3T3-L1 compared with 3T3 at all time points. Vice versa, TQ has a greater proliferative effect on 3T3 at higher concentrations compared with 3T3-L1 (Figures 1 and 2).

Percentage of viable cells computed in comparison with untreated cells is calculated as 100%. The value of IC_10_ is the mean from three independent experiments. Each value was represented as mean ± SEM.

### 3.2. Effect of TQ-NLC or TQ on the Mode of Cell Death of 3T3 and 3T3-L1

To further confirm the proliferative effect of TQ-NLC or TQ, analysis on the mode of cell death using annexin V assay was performed. Based on the IC_10_ values obtained from the MTT assay, three concentrations of TQ-NLC or TQ (3, 6 and 12 *μ*M) were selected. In 3T3, TQ-NLC showed no toxic effect with relatively high percentage of healthy cells (87–95%) and low percentage of apoptotic cells (<12%) (Table 2). Meanwhile, in 3T3 or 3T3-L1 treated with TQ (Tables 3 and 4), although not much different in the percentage of healthy cells compared with control, the percentage of necrotic cells was lower in almost all concentrations and time points.

Comparatively, the percentage of 3T3-L1 viable cells increased significantly with the increased concentration of TQ-NLC compared with TQ (Table 5). Treatment with TQ-NLC also reduced the number of necrotic 3T3-L1 in a time-dependent manner. At 3 *μ*M of TQ-NLC, only 10.1% of cells were confirmed to be categorized as apoptotic after 48 hours of treatment, and it decreased to 5.7% after 72 hours. Treatment with 6 *μ*M resulted in 8.6% of apoptotic cells at 24 hours and 11.9% of apoptotic cells in the control group. Meanwhile, NLC alone has almost similar percentage on viable, apoptotic, and necrotic cells for both 3T3 and 3T3-L1 as the control. Therefore, data obtained confirmed that TQ-NLC enhanced cell proliferation in both 3T3 and 3T3-L1 compared with control and TQ groups, and it is not influenced by its carrier.

### 3.3. Effect of TQ-NLC or TQ on Cell Migration

Wound healing assay was performed to see the migration effect of TQ-NLC or TQ on both cell models to cover the scratch created which mimics the wound. The distance was measured and analysed quantitatively using DinoEye 2.0 and ImageJ software at a time interval of 12, 24, and 48 hours after the scratch. Wound healing assay was made on serum starved medium by creating an artificial wound across the bottom of the culture plate followed by treatment with TQ-NLC or TQ. In both treated cell models, signs of cell migration were observed as early as 12 hours after treatment with TQ-NLC or TQ.

**Figure 3:**
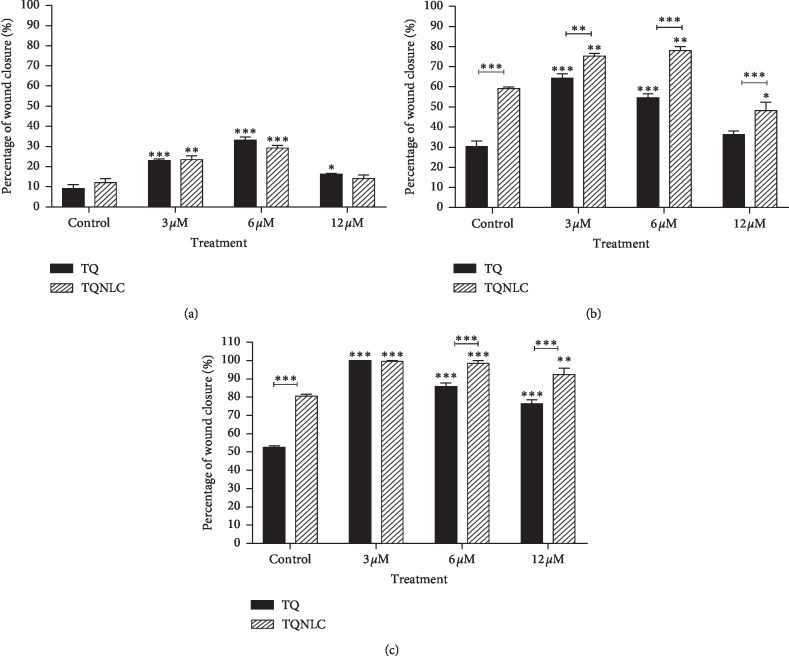
Bar graph of 3T3-L1 cells after treatment with TQ-NLC or TQ. A bar graph of TQ-NLC or TQ of 3T3-L1 fibroblast cells following treatment after (a) 12 h, (b) 24 h, and (c) 48 h as determined by scratch assay. The data are presented as mean ± SEM. Statistically significant differences between treated cells with control are indicated as ^*∗*^*p* < 0.05, ^*∗∗*^*p* < 0.01, and ^*∗∗∗*^*p* < 0.001. Statistically significant difference between TQ-NLC and TQ is also indicated in the graph. Data shown are the average of three independent experiments.

**Figure 4:**
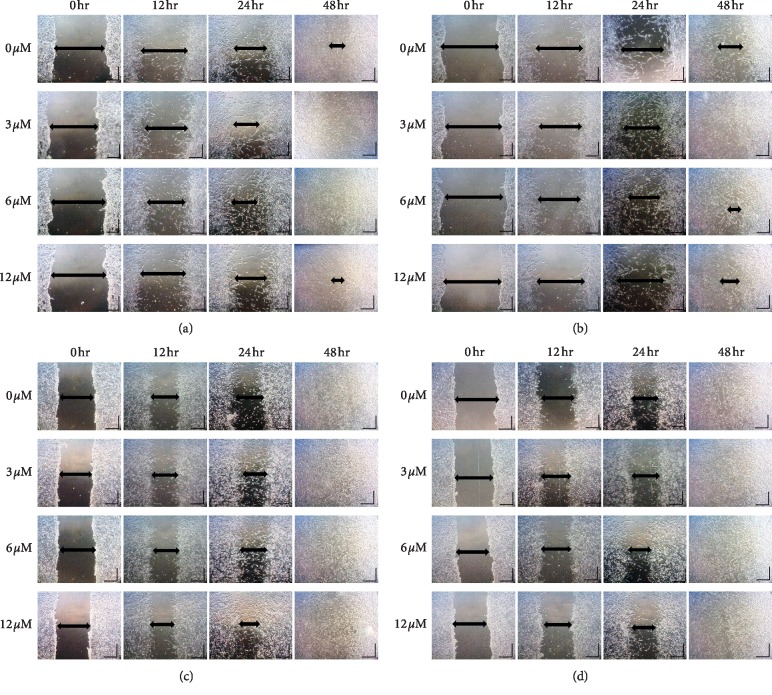
(a) Scratch assay images of serum-starved 3T3-L1 cells treated with TQ-NLC. The wound was completely closed at 3 *μ*M and 6 *μ*M concentration of TQ-NLC. At 24 hours, at least 50% of the gap was closed. The arrow shows the wound gap size. Wound closure rates are expressed as percentage of scratch closure after 48 hours compared with the initial area. Scale bar, 5000 *μ*M (100x magnification). (b) Scratch assay images of serum-starved 3T3-L1 cells treated with TQ. The wound was completely closed at 3 *μ*M concentration of TQ. At 24 hours, at least 50% of the gap was closed. The arrow shows the wound gap size. Wound closure rates are expressed as percentage of scratch closure after 48 hours compared with the initial area. Scale bar, 5000 *μ*M (100x magnification). (c) Scratch assay images of serum-starved 3T3 cells treated with TQ-NLC. The wound was completely closed at all concentrations of TQ-NLC at 48 hours. At 24 hours, at least 50% of the gap was closed. The arrow shows the wound gap size. Wound closure rates are expressed as percentage of scratch closure after 48 hours compared with the initial area. Scale bar, 5000 *μ*M (100x magnification). (d) Scratch assay images of serum-starved 3T3 cells treated with TQ. The wound was completely closed at all concentrations of TQ at 48 hours. At 24 hours, at least 50% of the gap was closed. The arrow shows the wound gap size. Wound closure rates are expressed as percentage of scratch closure after 48 hours compared with the initial area. Scale bar, 5000 *μ*M (100x magnification).

Although both compounds significantly enhanced the migration of 3T3-L1, a graph in Figure 3 shows that 3T3-L1 responded better towards the treatment of TQ-NLC as early as 24 hours with a significant difference observed when compared with TQ. Similarly, at 72 hours, TQ-NLC at 6 and 12 *μ*M shows better cell migration effect when compared with TQ and control groups. Figures 4(a) and 4(b) show the migration and proliferation of 3T3-L1 when treated with TQ-NLC or TQ, respectively.

**Figure 5:**
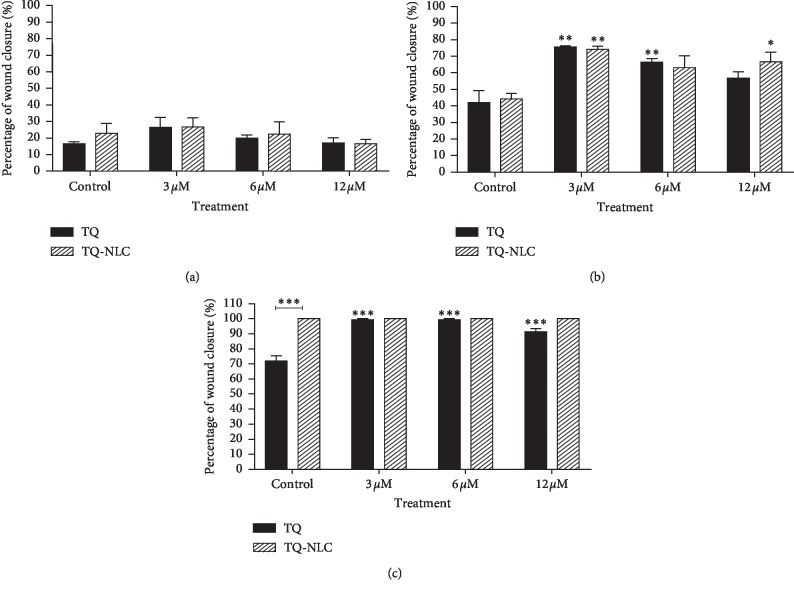
Bar graph of 3T3 cells after treatment with TQ-NLC or TQ. A bar graph of TQ-NLC or TQ of 3T3 fibroblast cells following treatment after (a) 12 h, (b) 24 h, and (c) 48 h as determined by scratch assay. The data are presented as mean ± SEM. Statistically significant differences between treated cells with control are indicated as ^*∗*^*p* < 0.05, ^*∗∗*^*p* < 0.01, and ^*∗∗∗*^*p* < 0.001. Statistically significant difference between TQNLC and TQ is also indicated in the graph. Data shown are the average of three independent experiments.

TQ also significantly promotes the migration of 3T3 (Figure 5). However, there was no significant difference in 3T3-treated TQ-NLC when compared with the control. Comparison of the migration effect between groups of TQ-NLC and TQ also did not reach statistically significant. Figures 4(c) and 4(d) show the cell migration and proliferation of 3T3-treated TQ-NLC or TQ. Thus, data obtained show that TQ-NLC efficiently enhanced the migration and proliferation of 3T3-L1 compared with normal 3T3. TQ-NLC also has a better migration effect compared with TQ and control groups.

### 3.4. Effect of TQ-NLC or TQ on the Intracellular ROS Production

**Figure 6:**
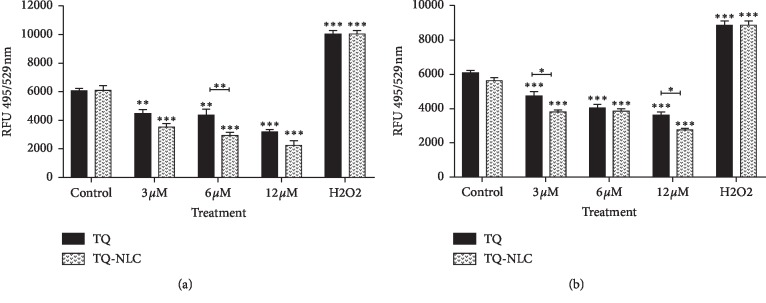
(a) ROS measurement in 3T3-L1 cell lines treated with TQ-NLC and TQ. The data are presented as mean ± SEM. Statistically significant differences are indicated with ^*∗*^*p* < 0.05. Data shown are the average of three independent experiments. (b) ROS measurement in 3T3 cells treated with TQ-NLC and TQ. The data are presented as mean ± SEM. Statistically significant differences are indicated with ^*∗*^*p* < 0.05. Data shown are the average of three independent experiments.

Figures 6(a) and 6(b) show that TQ-NLC or TQ is able to reduce the ROS level produced in both wounded cells compared with the control. Both of these compounds are able to exert their antioxidant activity at the concentration as lower as 3 *μ*M within 24 hours of treatment and as higher as 12 *μ*M without causing any harm towards the cells. In 3T3-L1 cells, TQ-NLC was observed to reduce more ROS compared with TQ and control groups with significant reduction observed at the concentration of 6 *μ*M of treatment. Similarly, TQ-NLC also has a greater antioxidant effect on 3T3 cells with its ability to significantly reduce the ROS level at the concentrations of 3 *μ*M and 12 *μ*M. Thus, data obtained show that despite the significant antioxidant effect showed by both compounds when compared with the control, TQNLC has a better antioxidant effect compared with its parental compound, TQ, on both cell models. Comparatively, H_2_O_2_ which acts as a marker significantly increased the ROS level in “wounded” cells when compared with the control.

## 4. Discussion

Cellular and biochemical events in wound healing play an important role in rearranging structural and functional continuity of the skin. Cell migration and proliferation are the most important steps and are thought to be the rate limiting factor in skin regeneration [54, 55]. When the precisely regulated steps of wound healing are disrupted, the normal wound healing process becomes impaired which is usually encountered in diabetic patients. It is attributed to several intrinsic and extrinsic factors such as neuropathy, wound infection, and trauma. Apart from being a metabolic disease, diabetes can also be considered to be an inflammatory disease. Inflammation enhances the progression of diabetes by decreasing peripheral insulin sensitivity [56].

The effectiveness of oral bioavailability of TQ is limited by its poor solubility and lipophilic nature in water [46, 57]. Hence, to overcome the disadvantages of TQ, thymoquinone-loaded nanostructured lipid carrier (TQ-NLC) was designed and effectively prepared by Ng et al. [49] via high-pressure homogenization technique. Although there is yet any report on the effect of TQ-NLC or TQ on wound healing in vitro and *in vivo*, TQ has been reported to be able to heal the burn wound in the rat model. However, there is no report postulating the effect of TQ-NLC on wound healing either in *in vitro* and *in vivo*. Due to the encapsulation of TQ into NLC, the drug efficiency increased, and the controlled drug release was improved. NLC is used for drug delivery via parenteral injecting, ocular, oral, pulmonary inhalation, and topical skin delivery [49, 58]. It is particularly useful for targeting water-soluble drug administration. For the ability to increase the solubility and improve oral bioavailability of poor lipophilic drug, lipid-based drug delivery is emerging as a promising oral carrier. Based on a study conducted by Ong et al. on acute toxicity, the encapsulation of TQ into NLC reduced the toxic effects of the compound which provides safety information on TQ-NLC [51].

In the current study, TQ-NLC or TQ was shown to specifically stimulate the fibroblast proliferation and migration which is considered an important factor for dermis regeneration [59]. Similarly, our IC_10_ values demonstrated that TQ-NLC or TQ was noncytotoxic towards normal fibroblast 3T3 cells. Both compounds were also noncytotoxic towards the mouse fibroblast mimic diabetic model, 3T3-L1. Although there has yet any report on the healing effect of TQ-NLC, both 3T3 and 3T3-L1 cell lines were commonly used in several cancer studies as comparison to assess the cytotoxicity of TQ [22, 60]. The IC_10_ value obtained supported the time course study where both 3T3 and 3T3-L1 treated with TQ-NLC had a lower IC_10_ value compared with TQ. Our data indicated that treatment of cells with various concentrations of TQ-NLC or TQ resulted in significant increase in cell viability compared with the control in both time- and dose-dependant manner.

Although the exact mechanism that causes this differential effect between TQ-NLC and TQ in still unknown, the encapsulated form of TQ may be one of the factors that contribute to this differential effect. The encapsulation of TQ with lipid carrier also minimizes the toxicity of the compound and improves its bioavailability. The presence of polysorbate 80 used in the formulation of TQ-NLC can be considered as an additive value to the TQ-NLC by enhancing the performance of TQ. Although not many studies were conducted to evaluate the toxicity of TQ-NLC, it is reported that TQ-NLC has a similar noncytotoxic characteristic as the parental compound towards the 3T3 cell line [57]. The antiapoptotic effect of TQ-NLC or TQ on normal cells had been reported in many studies [51, 61]. Similarly, our data confirmed the antiapoptotic activity of TQ-NLC or TQ on fibroblast cells by reducing the apoptotic cell number and increasing the number of healthy cells in 3T3 and 3T3-L1 cells. It is worth noting that both TQ-NLC or TQ did not promote necrosis in time and dose dependant manner in both cell models. The relatively low percentages of annexin V−/PI+ cells over dose and time course are suggested due to the release of proinflammatory intracellular contents.

Comparatively, TQ-NLC was observed to have better a proliferative effect than TQ with higher percentage of viable 3T3-L1. Although both compounds promote cell proliferation in 3T3 cells, the percentage of 3T3 viable cells was also higher when treated with TQ-NLC compared with TQ. Thus, the differential effect observed between TQ-NLC and TQ is consistent with the data obtained from MTT assay that show TQ-NLC has a better proliferative effect compared with TQ in both 3T3 and 3T3-L1. Proliferation effect by TQ-NLC also did not influence by its carrier since the percentage of both cells treated with NLC were similar as the control. The similar antiapoptotic effect on 3T3 was also observed when treated with TQ-NLC compared with TQ [57]. Therefore, TQ-NLC was confirmed to have better effect in promoting the proliferation of healthy cells and reducing the number of early apoptosis, late apoptosis as well as necrotic cells.

The strong proliferative and migration activity of TQ-NLC were reflected in the ability of the cells to increase the number of cells to cover the scratched wound areas. This assay mimics the third phase of wound healing, the proliferation phase, and consists of fibroblasts migrating and proliferating to close the wound [62, 63]. It is also important to note that increase in the proliferation and migration rate also depends on the nutrients provided. Therefore, to fully confirm that the proliferation of cells in this experiment is mainly due to the treatment and not influenced by the nutrients, serum was fully eliminated from the growth media provided for the both cells. Interestingly, both compounds were observed to enhance the cell migration as early as 12 hours with complete closure observed by 48 hours of treatment. Comparatively, TQ-NLC exerted its effect differently on both 3T3 and 3T3-L1 cell lines where the cell proliferation was generally higher compared with TQ.

Although the exact mechanism is yet to be known, it is postulated may be due to the encapsulated form of TQ that causes slow release with the focused effect in enhancing the proliferation of cells. Interestingly, as similar to the mode cell death data, our observation from the optimized data obtained show the migration effect by TQ-NLC was not influenced by its carrier. Thus, TQ-NLC may elicit its effect by enhancing the functional end point of fibroblast cell division and proliferation to migrate into the wounded area [64]. This causes the wounded cells to polarize towards the wounded area, migrate, and close the wound. In the *in vitro* wound healing process, fibroblast and keratinocyte cells migrate to the wound closure area forming the extracellular matrix [54]. Components of the extracellular matrix play regulatory roles in wound healing such as stimulating cell migration [65]. Collagen type I, mainly produced by fibroblasts to replace the temporary fibrin-based matrix, controls the dermal/epidermal cell adhesion and migration during skin restoration [4]. Considering all these facts, it may be speculated that TQ-NLC or TQ enhances the collagen production, which triggers proliferation and migration of fibroblast cells. A study of wound healing in the burn model shows that these anti-inflammatory and antimicrobial effects are responsible for accelerated wound healing capability of TQ [66].

It is inferred that the down regulation of inflammation during healing of the wound causes cell migration and proliferation to take place [67]. Meanwhile, the process of wound contraction depends on the angiogenesis process and the ability to repair damaged tissue, the condition of the tissue, and the extent of tissue damage [68]. To form cell-cell interactions, cells not only proliferate but also migrate by increasing the concentration of growth factors and cytokines in the wounded tissue. Through the influence of cytokines and chemokines, wounded cells are repaired extensively and immediately. It is also noted that cell injury may create higher oxidative stress environment. This condition may exhibit the turnover of healthy cells which is important for cell renewal and regeneration [69].

Reactive oxygen species (ROS) are essential for normal wound healing to take place. Through aerobic respiration and metabolism, about 2–5% of oxygen consumed by mitochondria generates ROS as a by-product [70, 71]. Numerous studies have pointed out that oxidative stress leads to chronic inflammation which in turn causes neurological and cardiovascular diseases. Superoxide anion radical (O_2_^−^·), hydroxyl radical (·OH), and peroxyl radical (ROO·) are a few types of ROS. They are continually generated by exogenous sources such as environmental pollution and UV radiation through aerobic metabolism. Oxidative stress is caused by an imbalance between ROS production and the cellular antioxidant defence system [72, 73]. Elevated levels of ROS that are continuously produced and sustained in cells are directly linked to impaired wound repair in chronic, nonhealing wounds [74].

Interestingly, the antioxidant effect of TQ-NLC or TQ has been reported to be significant in a cell-based system. In this study, TQ-NLC or TQ was seen to reduce the level of ROS produced in the cells at all concentrations of treatment given. Administration of TQ-NLC or TQ at all 3 dosages elicited marked antioxidant effects in both 3T3 and 3T3-L1 cell lines. The reduction of excessive ROS is important to maintain the healthy cell proliferation and speed the healing process. Interestingly, although 3T3-L1 is known to be a diabetic mimic cell model, TQ-NLC was able to significantly reduce the levels of ROS even at the lowest concentration of 3 *μ*M after 24 hours of treatment. TQ-NLC also showed a better effect compared with TQ although both compounds exert similar antioxidant effect in both cell lines.

Antioxidant enzymes are a vital part of the cellular defence against ROS. Wound healing mechanism may be caused by high antioxidant activity of the plant compounds or extracts used for its treatment. Antioxidants play an important role in the course of wound healing by decreasing the healing time and manifesting the healed tissue [75–77]. A proper and precise balance between the oxidative and antioxidative systems is vital for cell proliferation [78]. When a tissue is injured, a huge influx of ROS in the form of dismutated nonradical hydrogen peroxide or superoxide anion is released. They are important mediators of cellular signalling to commence the healing cascade, but an excess of ROS damages lipids, DNA, and proteins, hence affecting the proliferation and thus inhibiting the healthy cell turnover.

Despites the few research studies on TQ-NLC, it is hypothesized that the main TQ-NLC characters are still influenced by its parental compound, TQ. TQ is seen to have the scavenging activity against several ROS such as singlet molecular oxygen, superoxide anion, and hydroxyl radical [79–81]. Due to this, it is a very effective antioxidant and is able to antagonize the adverse effects resulting from elevated ROS levels in various disorders. Mansour et al. reported that TQ can act as a potent-free radical and superoxide radical scavenger at both nanomolar and micromolar ranges, respectively [79]. This was consistent with the report by Badary et al. that demonstrated TQ when administered in a dose-dependent manner, acted as a potent superoxide anion scavenger, and inhibited iron-dependent microsomal lipid peroxidation [82]. These results suggest that TQ is a radical scavenger with a potential role in the prevention and treatment of oxidative stress.

There are many previous studies, in accordance with the present study, that showed the ability of TQ to attenuate ROS generation. TQ was also shown to prevent the depletion of antioxidant defence mechanism and inhibit lipid peroxidation [83, 84]. It was also reported that TQ supplementation inhibits cyclooxygenase-2 (COX-2) expression which results in the reduction of inflammatory prostaglandins. It was also reported to decrease production of inflammatory chemokines and cytokines and suppress nuclear factor-κB inflammatory pathway [85, 86]. In addition, TQ was also reported to decrease production of nitric oxide (NO) and attenuate nitrosative stress by inhibiting the inducible nitric oxide synthase enzyme [87]. TQ exhibits strong antioxidant activity by upregulating superoxide dismutase (SOD), glutathione (GPX), and catalase (CAT) [88].

In the cellular culture, antioxidants may exert their effect exogenously or endogenously. They either scavenge free radicals or boost the intracellular antioxidant capacity [89]. Antioxidants help reduce the excess proteases and ROS formed by neutrophil accumulation and also protect protease inhibitors from oxidative damage. Overall antioxidant effects appear to be more important in successful treatment of wounds. One of the reasons that contributed to the reduction of the ROS levels in both groups of treated cells is the antioxidant capability of TQ. Therefore, our data established a novel finding on the improved bioavailability product, TQ-NLC and its parental compound, TQ, in 3T3-L1 and 3T3 cells which can be a promising wound healing agent. This study also suggests that TQ-NLC has a better effect in reducing the apoptotic cells and increasing cell proliferation as well as reducing the ROS levels at the wounded area. However, further studies are needed to explicitly explain the underlying mechanism of these actions.

## 5. Conclusions

Although there is yet any study to prove the ability of TQ-NLC or TQ to speed the proliferation migration rate of fibroblast cells, TQ or TQ-NLC showed a very minimum effect on growth inhibition in 3T3 and 3T3-L1 cells. TQ-NLC had a lower cytotoxic effect compared with TQ on both cell lines. TQ-NLC also confirmed to be a better candidate compared with TQ by enhancing the proliferation of both normal and diabetic cell models in a dose- and time-dependent manner. Interestingly, TQ-NLC promoted healthier cells with high percentage of viable cells and reduced the number of apoptotic as well as necrotic cells especially in the diabetic mimic cell model, 3T3-L1, compared with TQ. Although there is no explicit explanation for this, it is suggested that this may be due to the encapsulated form of TQ in NLC that contributed to the differential effect and improved its healing properties. TQ-NLC also promoted higher rate of migration in wounded cells especially in the 3T3-L1 cell and able to act as an antioxidant by reducing the amount of ROS in the wounded cells. Cells treated with TQ-NLC had a lower level of ROS compared with cells treated with TQ. Thus, TQ-NLC not only enhanced the proliferation and migration of cells but it was also able to act as an antioxidant to reduce the level of ROS in the normal and diabetic mimic cell model, while reducing the necrotic and apoptotic cell populations and increasing the number of healthy cells. This study concludes that TQ-NLC has the potential to be developed into a drug for treatment of diabetic wound.

## Figures and Tables

**Table 1:** Effects of TQ-NLC and TQ on normal fibroblast 3T3 and diabetic mimic 3T3-L1 reflected by the IC_10_ values using MTT assay.

Treatment	IC_10_ (*μ*M)					
	TQ-NLC			TQ		
Incubation time (hour)	24	48	72	24	48	72
3T3	4.3 ± 0.2	3.2 ± 0.5	2.5 ± 0.6	3.9 ± 2.1	5.9 ± 1.9	4.5 ± 1.4
3T3-L1	4.7 ± 3.3	3.5 ± 0.8	3.3 ± 0.5	5.3 ± 0.6	6.6 ± 1.1	5.3 ± 1.5

**Table 2:** Percentage of healthy, apoptotic, and necrotic cells of 3T3 treated with TQ-NLC as determined by flow cytometry.

		24 h	48 h	72 h
Control	Healthy cells	91.7 ± 0.1	89.4 ± 0.0	86.7 ± 1.2
	Early apoptosis	0.9 ± 0.1	1.2 ± 0.1	2.3 ± 0.6
	Late apoptosis	6.6 ± 0.1	8.7 ± 0.1	9.9 ± 0.6
	Necrosis	0.8 ± 0.1	0.7 ± 0.0	1.1 ± 0.1

NLC	Healthy cells	90.1 ± 0.2	89.9 ± 0.1	87.4 ± 0.0
	Early apoptosis	2.0 ± 0.1	2.2 ± 0.2	2.7 ± 0.3
	Late apoptosis	4.6 ± 0.1	4.5 ± 0.2	5.8 ± 0.3
	Necrosis	3.3 ± 0.1	3.4 ± 0.4	4.1 ± 0.1

3 *μ*M	Healthy cells	92.1 ± 0.8	89.3 ± 1.5	95.1 ± 0.8
	Early apoptosis	1.5 ± 0.3	0.9 ± 0.2	1.3 ± 0.2
	Late apoptosis	5.0 ± 0.6	8.3 ± 1.2	2.7 ± 0.4
	Necrosis	1.5 ± 0.4	1.5 ± 0.5	0.9 ± 0.2

6 *μ*M	Healthy cells	89.9 ± 0.2	92.8 ± 0.1	93.6 ± 0.9
	Early apoptosis	0.9 ± 0.1	0.6 ± 0.1	1.8 ± 0.1
	Late apoptosis	7.7 ± 0.3	4.9 ± 0.2	3.9 ± 0.1
	Necrosis	1.5 ± 0.0	1.8 ± 0.4	0.7 ± 0.7

12 *μ*M	Healthy cells	93.6 ± 2.0	94.7 ± 0.2	93.2 ± 0.7
	Early apoptosis	0.2 ± 1.1	0.5 ± 0.1	1.1 ± 0.1
	Late apoptosis	0.8 ± 2.4	3.8 ± 0.0	4.4 ± 0.6
	Necrosis	5.4 ± 1.5	1.0 ± 0.0	1.3 ± 0.0

Each data point represents the mean of three independent experiments. Each value was represented as mean ± SEM.

**Table 3:** Percentage of healthy, apoptotic, and necrotic cells of 3T3 treated with TQ as determined by flow cytometry.

		24 h	48 h	72 h
Control	Healthy cells	93.6 ± 0.4	91.4 ± 0.1	89.8 ± 0.6
	Early apoptosis	0.5 ± 0.1	1.6 ± 0.1	3.3 ± 0.8
	Late apoptosis	5.4 ± 0.4	5.5 ± 0.2	5.4 ± 0.1
	Necrosis	0.5 ± 0.1	1.5 ± 0.0	1.5 ± 0.1

3 *μ*M	Healthy cells	94.6 ± 0.4	93.7 ± 0.1	90.3 ± 0.6
	Early apoptosis	0.5 ± 0.1	1.2 ± 0.1	2.6 ± 0.4
	Late apoptosis	3.7 ± 0.7	3.4 ± 0.2	6.2 ± 0.2
	Necrosis	1.2 ± 0.3	1.7 ± 0.0	0.9 ± 0.0

6 *μ*M	Healthy cells	94.8 ± 0.7	91.4 ± 0.2	89.6 ± 0.7
	Early apoptosis	0.3 ± 0.1	1.0 ± 0.2	1.9 ± 0.2
	Late apoptosis	4.6 ± 0.4	6.6 ± 0.1	7.5 ± 0.9
	Necrosis	0.3 ± 0.2	1.0 ± 0.1	1.0 ± 0.4

12 *μ*M	Healthy cells	91.3 ± 0.4	90.2 ± 0.2	87.9 ± 2.1
	Early apoptosis	0.6 ± 0.0	1.2 ± 0.2	2.1 ± 0.3
	Late apoptosis	6.9 ± 0.2	7.3 ± 0.4	8.9 ± 1.9
	Necrosis	1.2 ± 0.2	1.3 ± 0.4	1.1 ± 0.1

**Table 4:** Percentage of healthy, apoptotic, and necrotic cells of 3T3-L1 treated with TQ as determined by flow cytometry.

		24 h	48 h	72 h
Control	Healthy cells	86.7 ± 1.8	84.7 ± 4.2	84.4 ± 2.4
	Early apoptosis	3.3 ± 0.1	1.1 ± 0.1	4.2 ± 0.4
	Late apoptosis	6.2 ± 0.8	10.3 ± 0.5	9.2 ± 1.7
	Necrosis	3.8 ± 0.9	3.9 ± 0.1	4.2 ± 0.3

3 *μ*M	Healthy cells	89.3 ± 0.3	80.6 ± 3.7	86.4 ± 2.1
	Early apoptosis	2.9 ± 0.3	1.2 ± 0.5	3.5 ± 0.6
	Late apoptosis	6.6 ± 0.4	15.3 ± 3.6	8.0 ± 1.3
	Necrosis	1.2 ± 0.4	2.9 ± 0.5	2.1 ± 0.3

6 *μ*M	Healthy cells	87.6 ± 2.1	82.5 ± 3.0	82.1 ± 3.1
	Early apoptosis	2.8 ± 1.1	1.2 ± 0.5	3.8 ± 0.5
	Late apoptosis	6.3 ± 1.2	13.1 ± 2.8	10.5 ± 1.2
	Necrosis	3.3 ± 0.6	3.2 ± 0.8	3.6 ± 0.3

12 *μ*M	Healthy cells	88.7 ± 1.3	84.1 ± 2.8	82.9 ± 2.9
	Early apoptosis	2.3 ± 0.8	1.9 ± 0.3	3.9 ± 0.7
	Late apoptosis	5.5 ± 0.6	11.5 ± 2.7	9.9 ± 1.9
	Necrosis	3.5 ± 0.6	2.5 ± 0.4	3.3 ± 0.6

**Table 5:** Percentage of healthy, apoptotic, and necrotic cells of 3T3-L1 treated with TQ-NLC as determined by flow cytometry.

		24 h	48 h	72 h
Control	Healthy cells	85.9 ± 1.6	88.5 ± 0.7	88.5 ± 0.7
	Early apoptosis	2.2 ± 0.3	1.0 ± 0.1	3.8 ± 0.3
	Late apoptosis	9.7 ± 1.4	7.1 ± 0.6	4.3 ± 0.4
	Necrosis	2.2 ± 0.5	3.4 ± 0.2	3.4 ± 0.9

NLC	Healthy cells	87.0 ± 0.4	87.1 ± 0.8	86.3 ± 0.4
	Early apoptosis	2.8 ± 0.8	2.7 ± 0.1	2.9 ± 0.2
	Late apoptosis	8.3 ± 0.4	8.5 ± 0.1	8.9 ± 0.2
	Necrosis	1.9 ± 0.1	1.7 ± 0.3	1.9 ± 0.2

3 *μ*M	Healthy cells	88.5 ± 1.2	86.4 ± 1.3	91.2 ± 2.0
	Early apoptosis	2.1 ± 0.7	1.5 ± 0.2	3.5 ± 0.2
	Late apoptosis	7.5 ± 0.7	8.6 ± 1.4	2.2 ± 1.7
	Necrosis	1.9 ± 0.4	3.5 ± 0.8	3.1 ± 0.0

6 *μ*M	Healthy cells	90.3 ± 0.4	92.4 ± 0.5	89.4 ± 0.9
	Early apoptosis	2.4 ± 0.2	0.9 ± 0.2	4.4 ± 1.3
	Late apoptosis	6.2 ± 0.5	3.7 ± 0.5	2.6 ± 0.4
	Necrosis	1.1 ± 0.0	3.0 ± 0.2	3.6 ± 0.4

12 *μ*M	Healthy cells	91.2 ± 0.2	92.6 ± 0.3	91.3 ± 2.1
	Early apoptosis	2.3 ± 0.4	0.7 ± 0.2	4.2 ± 1.4
	Late apoptosis	5.3 ± 1.1	2.5 ± 0.7	1.9 ± 1.4
	Necrosis	1.2 ± 0.7	4.2 ± 0.6	2.6 ± 0.5

## Data Availability

The data used to support the findings of this study are included within the article.

## Supplementary Materials

Supplementary Materials Figure 7. dose-response curve of (A) TQ and (B) TQ-NLC of 3T3 fibroblast cells. (A) TQ treated on 3T3 cells and (B) TQ-NLC treated on 3T3 cells following treatment after 24 h, 48 h, and 72 h as determined by MTT assay. The data are presented as mean ± SEM. Figure 8. dose-response curve of (A) TQ and (B) TQ-NLC of 3T3-L1 fibroblast cells. (A) TQ treated on 3T3-L1 cells and (B) TQ-NLC treated on 3T3-L1 cells following treatment after 24 h, 48 h, and 72 h as determined by MTT assay. The data are presented as mean ± SEM.
