# Supplementary material for: Migration and Proliferation Effects of Thymoquinone-Loaded Nanostructured Lipid Carrier (TQ-NLC) and Thymoquinone (TQ) on In Vitro Wound Healing Models
Source: Evid Based Complement Alternat Med. 2019 Nov 29;2019:9725738. doi: 10.1155/2019/9725738 (PMC6935463; doi:10.1155/2019/9725738)
Supplement: Supplementary Materials — Figure 7. dose-response curve of (A) TQ and (B) TQ-NLC of 3T3 fibroblast cells. (A) TQ treated on 3T3 cells and (B) TQ-NLC treated on 3T3 cells following treatment after 24 h, 48 h, and 72 h as determined by MTT assay. The data are presented as mean ± SEM. Figure 8. dose-response curve of (A) TQ and (B) TQ-NLC of 3T3-L1 fibroblast cells. (A) TQ treated on 3T3-L1 cells and (B) TQ-NLC treated on 3T3-L1 cells following treatment after 24 h, 48 h, and 72 h as determined by MTT assay. The data are presented as mean ± SEM. [file 9725738.f1.pdf]

Supplementary material.

A

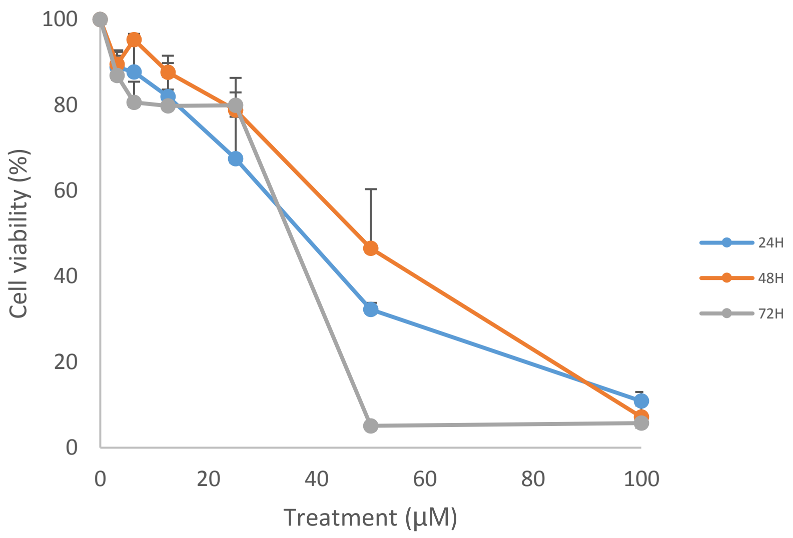


B

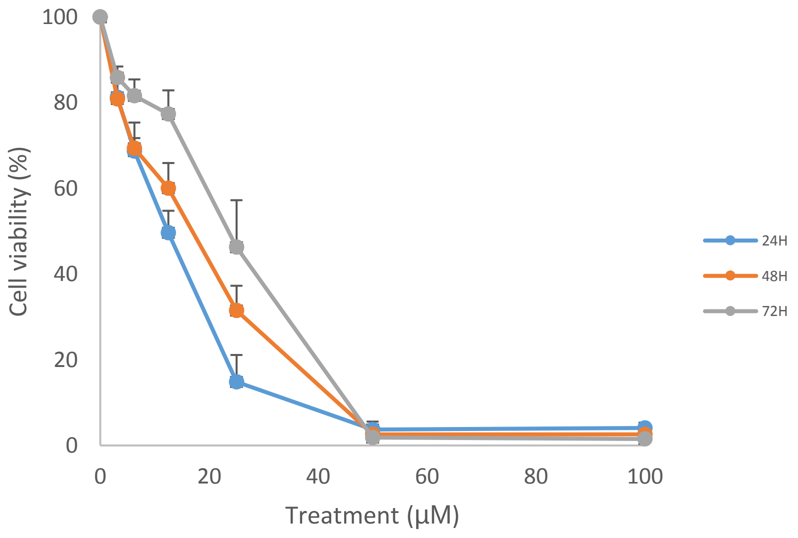


Figure 7. Dose-response curve of (A) TQ and (B) TQ-NLC of 3T3 fibroblast cells. (A) TQ treated on 3T3 cells and (B) TQ-NLC treated on 3T3 cells following treatment after 24 h, 48 h and 72 h as determined by MTT assay. The data are presented as mean ± SEM.

A

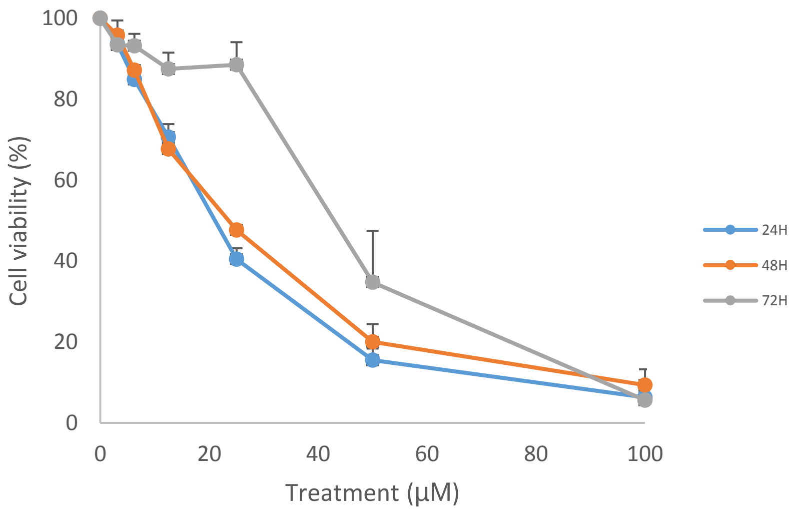


B

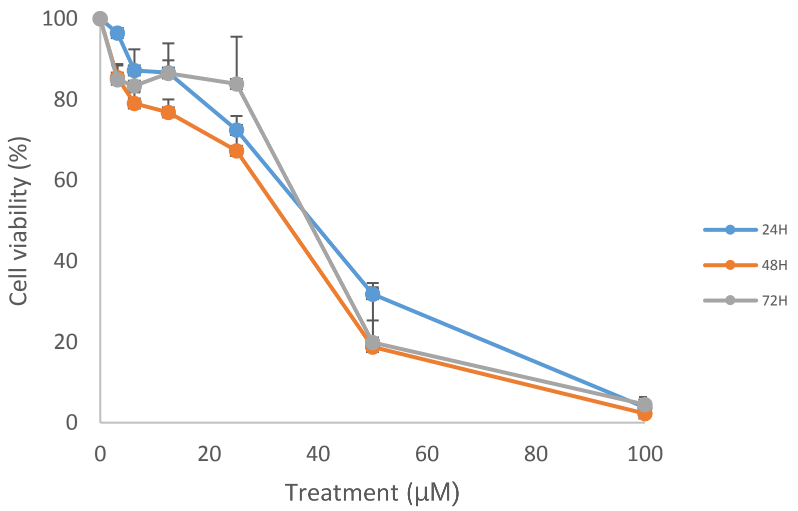


Figure 8. Dose-response curve of (A) TQ and (B) TQ-NLC of 3T3-L1 fibroblast cells. (A) TQ treated on 3T3-L1 cells and (B) TQ-NLC treated on 3T3-L1 cells following treatment after 24 h, 48 h and 72 h as determined by MTT assay. The data are presented as mean ± SEM.
